# Prioritizing disease-related genes and pathways by integrating patient-specific iPSC-derived RNA-seq and whole genome sequencing in hypoplastic left heart syndrome

**DOI:** 10.1186/1471-2105-15-S10-P7

**Published:** 2014-09-29

**Authors:** Xing Li, Almudena Martinez-Fernandez, Jeanne Theis, Jean-Pierre Kocher, Andre Terzic, Timothy Olson, Timothy J Nelson

**Affiliations:** 1 Division of Biomedical Statistics and Informatics, Department of Health Sciences Research, Mayo Clinic, Rochester, MN 55905, USA; 2 Division of Cardiovascular Diseases, Department of Medicine, Mayo Clinic, Rochester, MN 55905, USA; 3 Department of Molecular Pharmacology and Experimental Therapeutics, Mayo Clinic, Rochester, MN 55905, USA; 4 Division of Pediatric Cardiology, Department of Pediatric and Adolescent Medicine, Mayo Clinic, Rochester, MN 55905, USA; 5 Center for Regenerative Medicine, Mayo Clinic, Rochester, MN 55905, USA; 6 Transplant Center, Mayo Clinic, Rochester, MN 55905, USA; 7 Division of General Internal Medicine, Department of Medicine, Mayo Clinic, Rochester, MN 55905, USA

## Background

Hypoplastic left heart syndrome (HLHS) is a congenital heart defect in which the left ventricle of the heart is severely underdeveloped. Applying patient-specific induced pluripotent stem cells (iPSC) with high-throughput sequencing technology in RNA-seq and whole genome sequencing (WGS) provides an unprecedented opportunity to investigate the disease-specific transcription profiles linked to potential genetic causes in HLHS. Bioengineered HLHS patient-specific iPSCs and differentiated cardiac tissues offer a platform to recapitulate the individual developmental process to study the molecular causes of the disease.

## Materials and methods

In this study we reprogrammed the skin fibroblasts from proband and parents into iPSCs that were subsequently differentiated towards beating cardiomyocytes. We performed the RNA-seq on iPSCs and differentiated cardiomyocytes. Whole genome sequencing was done on blood samples from proband and parents. Combining expression differences between patient-specific cells with genomic mutations, we analyzed and integrated all these data to identify the potential genes related to HLHS.

## Results

We have identified 4000 and 6000 differential genes between the family members in iPSC and differentiated cells respectively. Most the differential genes show a high expression pattern in iPSC from proband. However, the pattern from differentiated cells showed both high and low expression in proband compared with parent. 40 genes with different types of mutations, including compound heterozygosity, X-linked, *de novo* mutations, were identified from whole genome sequencing data. LRP2 and PRTG were low expressed in proband in iPSC and differentiated cells and high expressed in the early heart developmental stages. However, DHCR24 were highly expressed in proband in both iPSC and differentiated beating cells and also highly expressed in the early stage of embryogenesis. Furthermore, MYLK, a later expressed gene in natural cardiogenic roadmap, is also highly expressed in proband.

## Conclusions

By integrating the data from WGS, RNA-seq, and the naturally expressed time-course developmental roadmap, we triangulated a list of prioritized candidate genes that may contribute to HLHS and could be a target for future mechanistic studies for disease-specific clinical applications.

